# Supplementary material for: Applying behavioural economics principles to increase demand for free HIV testing services at private doctor-led clinics in Johannesburg, South Africa: A randomised controlled trial
Source: PLOS Glob Public Health. 2024 Aug 6;4(8):e0003465. doi: 10.1371/journal.pgph.0003465 (PMC11302913; doi:10.1371/journal.pgph.0003465)
Supplement: S1 Fig — (DOCX) [file pgph.0003465.s002.docx]

**S1 Fig: HTS demand creation material***

Standard of Care Brochure Recipient of Care Voucher Healthy Lifestyle Screening Brochure


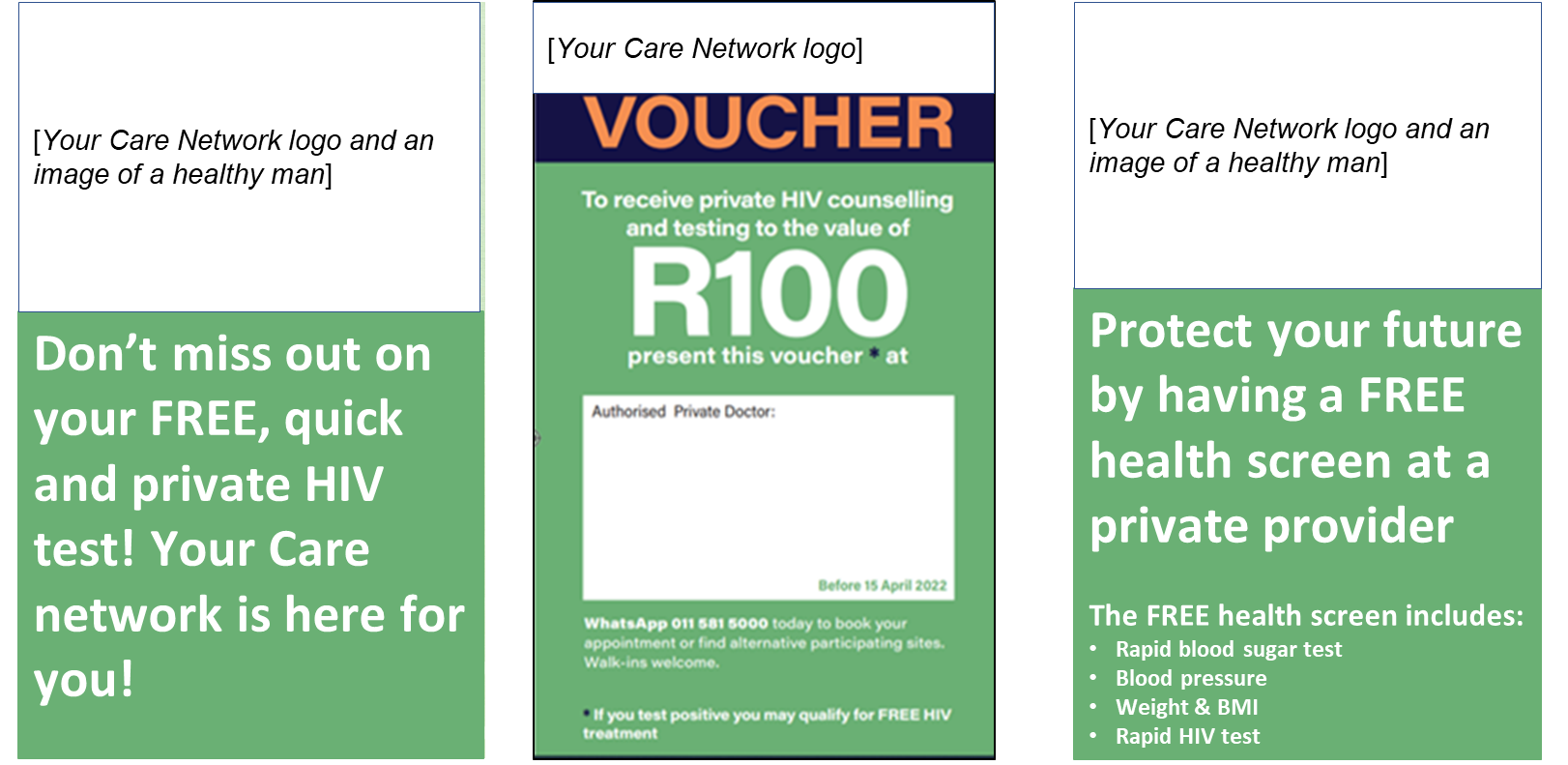


*The images on this flyer are not displayed due to copyright restrictions. These figures only include the text that was used as part of the brochures for the study.
