# Supplementary material for: Applying behavioural economics principles to increase demand for free HIV testing services at private doctor-led clinics in Johannesburg, South Africa: A randomised controlled trial
Source: PLOS Glob Public Health. 2024 Aug 6;4(8):e0003465. doi: 10.1371/journal.pgph.0003465 (PMC11302913; doi:10.1371/journal.pgph.0003465)
Supplement: S1 Table — (DOCX) [file pgph.0003465.s003.docx]

**S1 Table:** Sensitivity analysis of Odds ratios (95% CI) from exploratory logistic regression results comparing SOC, healthy lifestyle brochure and care recipient voucher brochure arms*

|  |  | **No. of participants** | **Presenting at the GP practice (%)** | **UOR (95% CI)** | **P-value** | **AOR (95% CI)**** | **P-value** |
| --- | --- | --- | --- | --- | --- | --- | --- |
| **Study arm** | SOC | 3603 | 137 (3.8%) | 1 [Ref] |  | 1 [Ref] |  |
|  | HLS | 3620 | 153 (4.2%) | 1.11 (0.88-1.40) | .394 | 0.92 (0.70-1.21) | .554 |
|  | RCV | 3608 | 158 (4.4%) | 1.12 (0.89-1.41) | .347 | 0.99 (0.76-1.23) | .924 |
| **Gender** | Male | 6406 | 227 (3.5%) | 1 [Ref] |  | 1 [Ref] |  |
|  | Female | 4298 | 221 (5.1%) | 1.11 (0.89-1.38) | .371 | 0.97 (0.77-1.21) | .762 |
| **Age group** | 18-24 | 1744 | 65 (3.7%) | 1 [Ref] |  | 1 [Ref] |  |
|  | 25-34 | 5438 | 215 (4.0%) | 0.76 (0.56-1.01) | .061 | 0.95 (0.70-1.28) | .728 |
|  | 35-44 | 2927 | 120 (4.1%) | 0.76 (0.55-1.05) | .101 | 0.89 (0.64-1.63) | .509 |
|  | ≥ 45 | 612 | 48 (7.8%) | 1.31 (0.84-2.04) | .230 | 1.04 (0.66-1.63) | .865 |
| **GP group** | GP group with limited visibility | 6630 | 44 (0.7) | 1 [Ref] |  | 1 [Ref] |  |
|  | GP group with high visibility | 4201 | 404 (9.6) | 6.49 (5.15-8.18) | .000 | 4.92 (3.82-6.34) | .000 |
| **Language** | English | 7756 | - | 1 [Ref] |  | 1 [Ref] |  |
|  | IsiZulu | 2466 | - | 1.50 (1.21-1.85) | .000 | 1.06 (0.82-1.37) | .665 |
|  | SeSotho | 609 | - | 1.57 (1.09-2.26) | .016 | 0.63 (0.33-1.20) | .163 |

* data excludes all duplicates

**Adjusted for study arm category, gender, age group, GP group and language
