# Supplementary material for: Applying behavioural economics principles to increase demand for free HIV testing services at private doctor-led clinics in Johannesburg, South Africa: A randomised controlled trial
Source: PLOS Glob Public Health. 2024 Aug 6;4(8):e0003465. doi: 10.1371/journal.pgph.0003465 (PMC11302913; doi:10.1371/journal.pgph.0003465)
Supplement: S2 Table — (DOCX) [file pgph.0003465.s004.docx]

**S2 Table:** Proportion of individuals presenting at the GP practice over the total number of brochures distributed by study arm

|  | | **Standard of Care** | | **Healthy Lifestyle Brochure** | | **Recipient Care Voucher** | |
| --- | --- | --- | --- | --- | --- | --- | --- |
|  |  | **Brochures distributed** | **Individuals presenting** | **Brochures distributed** | **Individuals presenting** | **Brochures distributed** | **Individuals presenting** |
|  | | n | n (%) | n | n (%) | n | n (%) |
| **Total** | | 3802 | 137 (3.6) | 3840 | 153 (4.0) | 3829 | 158 (4.1) |
| **Gender** | Male | 2246 | 69 (3.1) | 2293 | 76 (3.3) | 2235 | 82 (3.7) |
|  | Female | 1532 | 68 (4.4) | 1512 | 77 (5.1) | 1557 | 76 (4.9) |
| **Age group** | 18-24 years | 654 | 24 (3.7) | 612 | 17 (2.8) | 624 | 24 (3.8) |
|  | 25-34 years | 1901 | 67 (3.5) | 1911 | 60 (3.1) | 1962 | 88 (4.5) |
|  | 35-44 years | 1015 | 37 (3.6) | 1060 | 48 (4.5) | 991 | 35 (3.5) |
|  | ≥ 45 years | 216 | 9 (4.2) | 228 | 28 (12.3) | 225 | 11 (4.9) |
| **HIV test outcomes** | HIV negative | - | 130 (3.4) | - | 146 (3.8) | - | 156 (4.1) |
|  | PLHIV | - | 4/137 (0.1) | - | 5/153 (0.1) | - | 1/158 (0.0) |
|  | Unknown HIV outcomes | - | 3 (0.1) | - | 2 (0.1) | - | 1 (0.0) |
