# Supplementary material for: Applying behavioural economics principles to increase demand for free HIV testing services at private doctor-led clinics in Johannesburg, South Africa: A randomised controlled trial
Source: PLOS Glob Public Health. 2024 Aug 6;4(8):e0003465. doi: 10.1371/journal.pgph.0003465 (PMC11302913; doi:10.1371/journal.pgph.0003465)
Supplement: S3 Table — (DOCX) [file pgph.0003465.s005.docx]

**S3 Table.** Effect of intervention on odds of presenting at the GP practice with sociodemographics*

|  |  | **No. of participants** | **Presenting at the GP practice (%)** | **UOR (95% CI)** | **P-value** | **AOR 95% (CI)** | **P-value** |
| --- | --- | --- | --- | --- | --- | --- | --- |
| **Study arm** | SOC | 3802 | 137 (3.6%) | 1 [Ref] |  | 1 [Ref] |  |
|  | HLS | 3840 | 153 (4.0%) | 1.11 (0.88-1.40) | .384 | 1.02 (0.79-1.32) | .873 |
|  | RCV | 3829 | 158 (4.1%) | 1.15 (0.91-1.45) | .236 | 1.08 (0.84-1.40) | .559 |
| **Gender** | Male | 6774 | 227 (3.4%) | 1 [Ref] |  | 1 [Ref] |  |
|  | Female | 4601 | 221 (4.8%) | 1.11 (0.89-1.38) | .371 | 1.02 (0.82-1.26) | .852 |
| **Age group** | 18-24 | 1890 | 65 (3.4%) | 1 [Ref] |  | 1 [Ref] |  |
|  | 25-34 | 5774 | 215 (3.7%) | 0.82 (0.62-1.08) | .155 | 1.03 (0.77-1.37) | .856 |
|  | 35-44 | 3066 | 120 (3.9%) | 0.77 (0.56-1.06) | .108 | 0.90 (0.65-1.24) | .505 |
|  | ≥ 45 | 669 | 48 (7.2%) | 1.27 (0.83-1.94) | .274 | 0.98 (0.63-1.51) | .918 |
| **GP group** | GP group with limited visibility | 6885 | 44 (0.6%) | 1 [Ref] |  | 1 [Ref] |  |
|  | GP group with high visibility | 4586 | 404 (8.8%) | 6.13 (4.86-7.72) | .000 | 5.30 (4.14-6.79) | .000 |
| **Language**** | English | 8165 | - | 1 [Ref] |  | 1 [Ref] |  |
|  | IsiZulu | 2656 | - | 1.46 (1.18-1.80) | .000 | 0.98 (0.77-1.26) | .895 |
|  | SeSotho | 650 | - | 1.49 (1.03-2.16) | .035 | 1.07 (0.67-1.72) | .762 |

*Adjusted for gender, age group, clinic group, language (using combined GP groups and combined intervention arms)

**Language was not recorded at presentation at the GP practice
