# Supplementary material for: Applying behavioural economics principles to increase demand for free HIV testing services at private doctor-led clinics in Johannesburg, South Africa: A randomised controlled trial
Source: PLOS Glob Public Health. 2024 Aug 6;4(8):e0003465. doi: 10.1371/journal.pgph.0003465 (PMC11302913; doi:10.1371/journal.pgph.0003465)
Supplement: S1 Protocol — (PDF) [file pgph.0003465.s006.pdf]

Using behavioural science to increase demand for free HIV Testing Services at private General Practitioners (GPs) in the GP Care Cell (GPCC) network, Johannesburg Health District, Gauteng Province, South Africa

---

Short Title: Using behavioural science to increase demand for free HIV Testing Services at private General Practitioners

Protocol Version: 2.0 dated 03 January 2022

Funder: University of Pennsylvania, USA

Implementing Partner: Foundation for Professional Development (FPD), South Africa

Principal Investigator/Indlela Lead (UPenn): Dr. Harsha Thirumurthy

Co-Principal Investigator (FPD): Mrs. Suzanne J Mabaso

Co-Principal Investigator (HE^2RO): Dr. Candice Chetty-Makkan

Co-Investigator (HE^2RO): Dr. Sophie Pascoe

Co-Investigator (HE^2RO): Dr. Jacqui Miot

Co-Investigator (BU): Dr. Lawrence Long

UPenn Manager: Ms. Laura Schmucker

Nudge Associate: Ms. Simamkele Bokolo

IRB Federalwide Assurance numbers:

FWA00000715 (University of the Witwatersrand)

FWA00004028 (University of Pennsylvania)

FWA00000301 (Boston University)

Contents

1. **BACKGROUND** **6**
    - 1.1 The HIV Care continuum in South Africa: where are the gaps? 6
    - 1.2 The GP Care Cell (GPCC) programme 6
    - 1.3 Demand creation for the GP Care Cell programme 7
    - 1.4 Rationale / Purpose 7
    - 1.5 Study objectives 8
2. **METHODS** **9**
    - 2.1 Setting 9
    - 2.2 Study participants 10
    - 2.3 Study design and intervention 10
    - 2.4 Randomization procedures 11
    - 2.5 Recruitment procedures 11
    - 2.6 Study outcomes 12
    - 2.7 Sample size 13
    - 2.8 Process measures 14
    - 2.9 Data collection tools and data systems 14
    - 2.10 Data Analysis 15
3. **ETHICAL CONSIDERATIONS** **16**
    - 3.1 Informed consent 16
    - 3.2 Ethical review 17
    - 3.3 Study benefits 17
    - 3.4 Risks 18
    - 3.5 Study compensation 18
    - 3.6 Safety and adverse events 18
    - 3.7 Study administration, data handling and record keeping 18
    - 3.8 Study monitoring 20
4. **STUDY TIMELINE** **21**
5. **FUNDING SOURCES** **22**
    - 5.1 Funding source 22
    - 5.2 Conflict of interest 22
6. **DISSEMINATION PLAN** **22**
7. **REFERENCES** **23**

8. APPENDICES **23**

Appendix 1: Healthy lifestyle screen brochure 23
Appendix 2: Recipient of care voucher 23
Appendix 3: YCN standard of care brochure 23
Appendix 4: YCN field worker script for recruitment 23
Appendix 5: Distribution log 23
Appendix 6: Screening and enrolment log 23
Appendix 7: GPCC HTS consent form 23
Appendix 8: Participant information sheet 23
Appendix 9: Participant written informed consent form 24

Acronyms

ART: Antiretroviral therapy

FPD: Foundation for Professional Development

HIV: Human Immunodeficiency Virus

HTS: HIV Testing Services

YCN: Your Care Network

Study Summary

Background: Increasing the utilization of HIV services among people living with HIV (PLHIV) and individuals at high risk of HIV exposure is essential for ending the HIV epidemic in South Africa. There is low uptake of HIV testing services (HTS) by uninsured people at the contracted General Practitioner Care Cell (GPCC) sites who provide HIV tests. The GPCC is a well-functioning National Health Insurance (NHI)-aligned demonstration programme which aims to expand access to HIV testing services (HTS) and antiretroviral therapy (ART) for uninsured PLHIV by leveraging the capacity of private general practitioners (GPs) outside of public sector health facilities in a controlled manner. There is a social media campaign called Your Care Network (YCN), but it is not sufficient for promoting utilization of HIV services at GP practices that are nearby individuals' homes and workplaces. Although the GP care cell (GPCC) programme offers a standardized Your

Care Network (YCN) demand creation package, GPs are expected to generate HTS demand independently. Many GPs do not invest in community demand creation at their practices as the immediate benefits to the practice are not clear and they have limited expertise in community outreach. In addition, individuals may not be aware that GPs who participate in the GPCC programme offer free HIV testing services. By using behavioural insights to promote the availability of free HIV services, it may be possible to increase uptake of HTS at GP practices.

Objectives: To determine whether HTS demand creation material that leverages behavioural science principles will increase the demand for HIV testing at GP practices.

Approach: A randomized controlled trial to rapidly test two different low-cost demand creation interventions among adults residing in the catchment areas of GPCC programme clinics. For one group of participants, free HIV testing will be bundled with other health services that might be desired. Participants will receive a brochure that advertises the opportunity to receive free blood pressure testing and rapid glucose testing along with free HIV testing. This approach seeks to address stigma associated with HIV testing while also offering other health services that people value. For a second group of participants, the value of HIV testing will be emphasized using a 100 Rand voucher (US$ 7) that can be redeemed for free services. This approach highlights the monetary value of the free services and seeks to leverage an endowment effect and loss aversion. A third group of participants will receive the standard of care promotion materials for HIV testing. The primary outcome will be presentation at the GP practice. Secondary outcomes will include HIV testing uptake, yield of new HIV-positive diagnoses, and yield of PLHIV who initiate ART. The study will be conducted at approximately five YCN GP practices in the Johannesburg Health District. We will seek to enrol 12, 000 adults in the vicinity of the GP practices who are willing to engage with a YCN field worker and willing to accept an envelope that contains one of the three HIV testing promotion strategies. The study duration will be approximately 10 months.

Significance: The standard marketing material (YCN brochure) and the YCN social media campaign have had limited success in increasing demand for HIV services offered by GP practices. There is a growing body of evidence from behavioural science showing that individuals' decision-making can be affected by the way in which information is presented and services are offered. We seek to test low-cost, easy-to-implement, demand creation strategies that can be implemented by the GPCC to increase demand for HIV services at GP practices.

1. BACKGROUND

1.1 The HIV Care continuum in South Africa: where are the gaps?

South Africa has built the world's largest HIV treatment program since starting to provide antiretrovirals in 2004. In 2020, there were an estimated 7.6 million people living with HIV (PLHIV) and 5.2 million (70%) were receiving antiretroviral therapy (ART) (1). Yet South Africa, like many countries, did not manage to reach the 90-90-90 targets by 2020 and is unlikely to reach the 95-95-95 targets by 2030 without further progress in expanding the utilization of HIV services. According to UNAIDS, 92% of PLHIV South Africans knew their status in 2020 but of those only 70% initiated treatment and of those 64% were virally suppressed (1). The General Practitioner Care Cell (GPCC) is an innovative, public-private partnership that seeks to increase coverage of ART among PLHIV by leveraging the resources in the private healthcare sector to reach uninsured individuals who are not accessing care.

1.2. The GP Care Cell (GPCC) programme

The General Practitioner Care Cell (GPCC) is a well-functioning National Health Insurance (NHI)-aligned demonstration programme which aims to expand access to HIV testing services (HTS) and antiretroviral therapy (ART) for uninsured people living with HIV (PLHIV) by leveraging the capacity of private general practitioners (GPs) outside of public sector health facilities in a controlled manner. The GPCC is a structured network of GP practices and community pharmacies that are organised and managed to deliver HIV testing and treatment services (HTS) using the purchaser-provider split as envisioned under the NHI. The goal of this programme is to implement a private sector contracting model that achieves good HIV patient outcomes and that is rapidly scalable, highly compliant with governance criteria, low risk and a relatively low administrative burden for government and the contracted service providers. The GPCC has been operational in Gauteng Province since May 2018.

The GPCC is funded by Anova Health Institute through USAID/PEPFAR funding for

implementation in Johannesburg Health District and has been prescribed ambitious HIV Testing Service (HTS) and antiretroviral treatment (ART) initiation targets for year one as a prerequisite for securing longer-term funding. The GPCC offers a contracting solution for (1) clinical management, (2) network management, (3) stock logistics and control, (4) financial administration, and (5) M&E. The programme currently offers HTS and ART within a private healthcare setting (GP practice) to individuals for no charge at the point of care; costs of the programme are covered by PEPFAR and drugs and laboratory tests are provided by the South African National Department of Health. This has been shown to be an extremely effective way of delivering HIV treatment, but it relies on the GP practices being able to have access to the appropriate patient population and getting new individuals into the practice to test.

1.3 Demand creation for the GP Care Cell programme

Currently, the GPCC offers no standardized demand creation package and GP practices are expected to create demand for these covered services themselves. There is a social media campaign called Your Care Network (YCN), but it is not sufficient for promoting utilization of HIV services at GP practices that are nearby individuals' homes and workplaces. Based on experience by the Foundation for Professional Development (FPD), contracted private sector GPs are not accustomed to mobilizing demand for their services at the community-level. The GPCC program is new and largely unknown by communities in the GPs' catchment areas, and without a successful, feasible practice demand creation strategy, the programme will not be able to reach scale.

1.4 Rationale / Purpose

The standard marketing material (YCN brochure) and the YCN social media campaign have shown limited yield in driving demand for services to the GP practice. It is possible that GPs do not invest in community demand approaches at their practices, as they may not see the immediate private good of providing HTS that may have long term consequences for individual well-being. In addition, individuals may not be aware that GPs offer free HIV testing services at their practice.

There is a vital need to develop low-cost, easy-to-implement, demand creation strategies that can increase demand for subsidized HIV services at GP practices. The interventions we propose to test in this study are motivated by behavioural science research showing that low-cost interventions that alter the way in which information is presented and services are offered can influence decision-making (2).

One insight that we leverage is that behaviours that individuals may be reluctant to undertake (e.g. HIV testing) can be motivated by bundling them with other services or items that people value. Thus we propose to offer individual free health screening that includes HIV testing as well as blood pressure and glucose screening. This also overcomes stigma associated with seeking HIV services. Another insight we use is that individuals may place have greater demand for an item or service if the true value of it is made salient and they are then given an opportunity to receive it for free. Thus, we propose to offer individuals HIV testing services in the form of a voucher that indicates the value of the service and provides a discount that makes the service free. We will compare the approaches to promoting demand for HIV services at GP practices to the standard YCN brochure that is being used to create demand. This study will be conducted in compliance with the protocol, applicable regulatory requirements, and respective IRB (Wits HREC, UPenn, BU) policies and procedures.

1.5 Study objectives

To determine whether HTS demand creation material that leverages behavioural science principles will increase the demand for HIV testing at GP practices.

The primary outcome will be presentation at the GP practice. Secondary outcomes will include HIV testing uptake, yield of new HIV-positive diagnoses, and yield of PLHIV who initiate ART.

This will be a randomized controlled trial to rapidly test two different low-cost demand creation

interventions among adults residing in the catchment areas of GPCC programme clinics. There will be three study groups:

1. For one group of participants, free HIV testing will be bundled with other health services that might be desired. Participants will receive a brochure that advertises the opportunity to receive free blood pressure testing and rapid glucose testing along with free HIV testing (Appendix 1). This approach seeks to address stigma associated with HIV testing while also offering other health services that people value.
2. For a second group of participants, the value of HIV testing will be emphasized using a 100 Rand voucher (US$ 6.67) that can be redeemed for free services (Appendix 2). This approach highlights the monetary value of the free services and seeks to leverage an endowment effect and loss aversion.
3. A third group of participants will receive the standard of care promotion materials for HIV testing. The standard of care (SOC) community demand procedure includes distributing a Your Care Network (YCN) brochure that advertises free HTS and free ART services for those that test positive (Appendix 3), a website (www.yourcarenetwork.co.za), a YCN Facebook page and standard signage and posters outside participating GP practices.

We will examine the effect of demand creation interventions on the likelihood that individuals will visit GP practices and utilize HIV services. We will also examine the effect of the interventions on identification of newly diagnosed PLHIV.

2. METHODS

2.1 Setting

The study will be conducted at GP practices in the Johannesburg Health District that are part of the GPCC. The Johannesburg Health District consists of seven sub-regions. We will purposively select approximately five GP practices that meet the following criteria:

(1) enrolled in the existing GP Care Cell/YCN programme;

(2) have a low performance in HIV testing and linkage to care;

(3) have a large catchment population to reach individuals eligible for HIV testing and linkage to care;

(4) office space to accommodate an HTS counsellor; and

(5) willingness to participate in the study.

Table 1 is a list of GP practices that includes the estimated sub-region population and estimated HIV prevalence per sub-region. YCN field workers are currently working in these sub-regions for the Johannesburg Health District.

Table 1: Purposively selected GP practices in the Johannesburg Health District

**Area**	**Region**	**Sub-region**	**Est. sub-region pop**	**Est. PLHIV** Prov. Estimate (12%)
Johannesburg CBD	F	60	39 405	4 729
Soweto	D	37	30 072	3 609
Johannesburg CBD	F	124	43 067	5 168
Hillbrow	F	60	39 405	4 729
Midrand	A	110	37 331	4 480

2.2 Study participants

Inclusion criteria: YCN field workers will approach individuals in the catchment areas of GP practices who meet the following criteria:

- Adults (≥18 years old)
- Willing to engage with the field worker for a few minutes to learn about GPCC
- Willing to accept an envelope containing promotion materials

Exclusion criteria:

- Individuals who refuse to engage with an YCN field worker

2.3 Study design and intervention

We will conduct a randomized control trial to determine the effectiveness of the new demand creation materials in reaching new individuals for HIV testing and linkage to care at GP practices. Individuals will be randomized to one of the study arms and will each receive an envelope containing the relevant demand creation material for their arm. The YCN field workers will use the recruitment script (Appendix 4) to inform individuals about HIV testing services at the GP practice and distribute the envelopes in the community. The name of the GP and contact number of the GP practice will be stamped on each brochure. The three study groups will include:

Study Group 1: A brochure promoting a free healthy lifestyle screen which includes HIV testing. This brochure will offer individuals options for a healthy lifestyle screen (weight, height, blood pressure, rapid glucose and HIV test). This brochure will be in addition to the standard of care demand creation material.

Study Group 2: A patient voucher for HTS (and ART): A voucher redeemable for free services valued at ZAR100 (USD 7) for HTS and approximately ZAR5,048 (USD 337) for ART (based on eligibility). The voucher will emphasize the value of the services offered. This brochure will be in addition to the standard of care demand creation material.

Study Group 3: Your Care Network (YCN) brochure advertising free HTS and free ART (for those who test positive) and the name and telephone contact number of the GP practice. This approach also includes existing social media platforms.

The healthy lifestyle and the patient voucher brochures will be co-designed with the study team and key stakeholders. The study demand creation material will be prototyped as part of the existing routine GPCC programme. Revisions to the brochures will be made prior to study implementation. The brochures will be submitted to ethics for approval prior to study implementation.

2.4 Randomization procedures

To test whether the two approaches are more effective than the standard of care approach in reaching new individuals for HIV testing and linkage to care at GP practices, individuals will be randomized in a 1:1:1 ratio to the three study arms. We will use computer-generated random numbers with stratification on study groups to get an equal number of study groups. After randomisation, brochures related to the specific study groups will be placed in different envelopes. Each envelope will be marked, entail one of three brochures and allocated in advance to the YCN field workers for distribution.

2.5 Recruitment procedures

Each YCN field worker will use a recruitment script (Appendix 4) to engage with potentially eligible individuals and will then hand them a marked envelope. Individuals will be recruited individually or in groups. The handing out of brochures is a standard demand creation strategy and no informed consent will be obtained from individuals during this interaction. The field worker will only record basic demographics (age group, gender) on individuals who receive the study envelopes on the REDCap system. This will be completely anonymised and not linked to any personal identifying information and only used to determine aggregate statistics. YCN field workers will target areas in the GP's catchment area (or within a local taxi ride) specifically targeting high volume and high transit settings. Some recruitment sites will include the industrial zone/formal workspaces, informal workspaces (trading stalls, spaza's, salons, veggie stalls etc), retail/shopping zones, establishments that sell food, educational settings (TVET, university, trade schools, etc), commuter stops and other community settings (indoor and outdoor venues). We will be able to record which envelopes are distributed at the different recruitment sites on REDCap using the data fields on the distribution log (Appendix 5).

Individuals who are interested in the services provided by the GP and attend the GP practice will be asked about how they heard about the practice and requested to hand-in the brochure that they received. The individual will then be requested to sign a study consent form (Appendix 8 and

9) before any data is collected and sign an HTS consent form (Appendix 7) before any medical procedures. As part of the research study and routine procedures at the GP practice, we will ask participants some questions. Questions will include but are not limited to demographic information (age, gender, telephone number and primary home address) and HTS outcomes (Date of HIV test, HIV test result and ART start date).

2.6 Study outcomes

The primary outcome for this study will be presentation at the GP practice. For each adult who is enrolled in the study and given information about the GPCC, we will determine whether they presented at one of the GP practices.

Secondary outcomes will include the following:

- HIV testing uptake: We will assess whether participants complete HIV testing at GP practices. This outcome will be defined for all adults who are enrolled in the study.
- HIV-positivity among participants who get tested for HIV.
- Linkage to care among newly diagnosed individuals. We will assess whether participants who tested HIV-positive initiate ART at the GP practices.

2.7 Sample size

We anticipate that about 5% of participants in the standard of care group will present at the GP. This is a conservative assumption based on the experiences of similar campaigns in which individuals are approached in the community. With 2,400 adults randomized to each of the three study arms (total sample size of 7,200 adults), we will have >80% power (alpha=0.05, 2-sided) to detect a difference of at least 2 percentage points in attendance at GP practices. However, since there is a possibility that a larger proportion of participants in the standard of care group will present at the GP, we have selected a sample size of 4,000 adults per arm (12,000 total) as this will ensure there is 80% power to detect a difference of at least 2 percentage points. A small difference in the primary outcome would be meaningful given the low cost of the demand creation interventions. With 5 field workers distributing materials over a period of 10

weeks,), the target of 12,000 adults reached by the field workers can be achieved by enrolling 48 individuals per day (which is feasible given the small amount of time needed to enrol an individual). HTS services at the GP practice will also start at the same time as the field work but will run for a period of 12 weeks. Provision of services at the GP practices will continue for an extra 2 weeks following the end of field work in order to allow individuals with the marketing material to still access HTS.

2.8 Process measures

The study aims to rapidly measure whether demand creation framed according to behavioural economics principles will increase HIV testing and related services at GP practices.

Process measures will include:

1. **Distribution of envelopes containing brochures of the different study groups in the community by the YCN field worker**
    - Number of envelopes distributed in the community, by study group
    - Demographics captured by the field worker of the target population that accepted the envelopes (age, gender)
2. **Number of people attending the GP practice**
    - Demographics captured by the GP practice of the target population that attends the GP practice for HTS services (age, gender), by study group
    - The following category of individuals attending the GP practice will be monitored:
        - Individuals that have a brochure & self-reports that they were recruited by a YCN field worker
        - Individuals that do not have a brochure but self-reports that they were recruited a YCN field worker using demand creation material from a specific study arm
        - Individuals that have the brochure & self-reports that someone else (friend/family) gave it to them

2.9 Data collection tools and data systems

Data will be collected and captured electronically by fieldworkers and HTS counsellors on the REDCap system. The data collection tools listed below will be built into the REDCap system. However, the study informed consent form and the HTS consent form will be completed on the paper-based form. All information relating to the participants' HIV and ART initiation status will only be captured on the Intelligent Care System (ICS). Below we provide a description of all the tools that will be captured both electronically and on paper.

- **Distribution log** (Appendix 5): This log will be used to track the distribution of envelopes in the community by the YCN field worker.
- **Participant consent for study participation** (Appendices 8 & 9): Individuals that present at the GP practice will be invited to participate in the study and interested participants will sign a written informed consent on a paper version.
- **Consent form for HTS procedures** (Appendix 7): As part of routine care, informed consent is obtained from all individuals before an HIV test is conducted. The HTS consent forms are the primary data collection source for recording HIV test results. Individuals interested in taking an HIV test will sign a paper version of the HTS consent form.
- **Screening and enrolment log** (Appendix 6): The screening and enrolment log will be completed on REDCap after the participant signs the research and HTS consent forms. In order to adhere to the Department of Health requirements for HIV testing, the identity numbers or date of birth of individuals who want to access HTS will be captured on REDCap. After the study consent form (Appendix 8 & 9) and the HTS informed consent (Appendix 7) are signed the individual's information will be captured onto the Intelligent Care System (ICS). The ICS system will then generate a unique identifier. Once the ICS-generated unique number is generated and the screening and enrolment log are captured electronically on REDCap, the individual's identity number or date of birth will be removed from the data set and the ICS unique number will serve as the participant study number.
- **Intelligent Care System (ICS):** ICS is the existing GPCC patient record platform used to record demographics of all patients enrolled in the GPCC program including those tested

for HIV and those started on ART. HTS is captured onto ICS from the HTS consent forms and the GP's clinical files.

- **Study data set:** Study data will be constructed based on merged electronic data sets from REDCap and the ICS data extract using the ICS unique number.

2.10 Data Analysis

Primary analysis: We will compare the primary outcome between study arms. We hypothesize that presentation at GP practices will be higher in the two intervention arms than in the standard of care arm. We will test for significant differences in the primary outcome between each of the intervention groups and the standard of care group using logistic regression analyses.

Secondary analyses: We will also compare the secondary outcomes in each intervention group to the standard of care group using logistic regression analyses. Our hypothesis is that HIV testing uptake will be higher in the intervention groups than the standard of care group.

3. ETHICAL CONSIDERATIONS

3.1 Informed consent

We request that informed consent be waived for individuals who are approached by the YCN field worker in the research based on the following:

The demand creation activities involve no more than minimal risk to subjects:

- This request for an informed consent waiver is only applicable to the individuals that the YCN field worker approaches in the field for the purposes of handing out a brochure
- During the field interaction, no personal identifying information will be collected. The distribution log (Appendix 5) that will be completed by the field worker on REDCap when interacting with an individual does not collect any identifying information. This information will only track recruitment indicators from the field
- Demand creation via printed material is a routine activity that is being implemented

already and the study poses no more than minimal risk. Individuals will all receive printed information about the services available to them, but the decision to present for those services is at the choice of the individual

- The demand creation activities involve no more than minimal risk to subjects.
- The demand creation activities could not be carried out practically without the waiver or alteration
- The waiver or alteration will not adversely affect the rights and welfare of the subjects.
- Where appropriate, the subjects will be provided with additional information about their participation
- Individuals who accept the brochure and attend the GP practice to access health services or HIV testing will be invited to sign a written informed consent. Consent will be obtained for study participation and HIV testing.

We will obtain written informed consent from the individuals that present for services at the GP practice for enrolment into the study, in order to extract the necessary data from their medical records and report on the primary and secondary outcomes (Appendix 8 & 9). Consent will be obtained for those attending the GP practice to assess medical records. Following standard GPCC practice when participants present for HTS they will be consented for the medical procedure (Appendix 7).

3.2 Ethical review

The protocol, informed consent forms, other requested documents and any subsequent modifications will be reviewed and approved by the University of Witwatersrand, University of Pennsylvania and Boston University Institutional Review Boards (IRBs) prior to starting this study. We will also obtain approval from the National Health Research Database (NHRD) for district approval. Subsequent to initial review and approval, the ethics board will review the study at least annually. These reports will include changes in the research activity and unanticipated

problems involving risks to study participants or others.

3.3 Study benefits

By taking part in this study, participants will not receive any direct benefits. However, participation in this research could benefit the community as a whole. In this research we will be able to find which are the best ways to give information to individuals that will help to encourage them to seek out and access health services including HIV care and treatment.

3.4 Risks

It is possible that the individuals' visit to GP practice and involvement in the associated research study could become known to others. This could result in social harms (i.e. where others may think that they are HIV-infected or at "high risk" for HIV infection). However, we will take special steps to maintain confidentiality and minimize the risk of social harm. Even though we have systems in place to protect the identity of individuals, there could be accidental disclosure of health data. Some individuals could also experience discomfort during the medical procedures. Individuals that take up the offer of free services may be diagnosed with HIV or other lifestyle conditions. This may cause the individual distress but ultimately early diagnosis allows for treatment and better outcomes.

3.5 Study compensation

Participants in the study will not be provided compensation for taking part in the study.
Participation in the study will be voluntary and participants can opt-out at any point.

3.6 Safety and adverse events

There are no known safety or adverse events of this study. Standard patient care will be followed when individuals access HIV testing services at the GP practice.

3.7 Study administration, data handling and record keeping

Study implementation will be directed by this protocol as well as study-specific procedures.

Standard operating procedures will outline more detailed procedures for conducting this study. Monthly meetings/teleconferences with investigators will occur to review study progress with corrective feedback supplied as necessary to implement the protocol.

We will collect routine demographic information (age, gender, telephone number and primary home address) and HTS outcomes (Date of HIV test, HIV test result and ART start date) from participants who sign the informed consent. We will ask participants these questions to follow up on their results and request permission to access their medical records. As soon as we have the data that we need, we will remove all personal identifiers. No identifiers will be included in any of the data that is analyzed or included in any results.

All confidential patient information regarding HIV status and antiretroviral treatment status and ID will be recorded in the Intelligent Care System (ICS), the GPCC's existing IT platform that includes detailed clinical care plans which are built on the Standard Treatment Guidelines and the Essential Medicine List (EML) and the GPCC-approved medicines and laboratory formularies. The ICS manages all data linked to the individuals/patients (demographics, screenings, clinical care, laboratory services, prescription and dispensing activities) and engagement (date and time) in a secure environment and is implemented real time or near-real time based on paper-based source documents (e.g. HTS consent forms etc.). As part of the GPCC Standard of Care, data are reviewed and verified on a weekly basis with routine random samples to verify patients and data accuracy to source documentation. The ICS system currently generates all data needed for clinical management and routine reporting for the GPCC program and will be used to capture and store HTS screening and ART data for analysis under this research project.

All data are encrypted with password protection, allocated roles and responsibilities per user. Data are longitudinal and linked to named/identified patients and will allow for sophisticated data analysis and summary in line with research requirements. Data extracts are restricted in line with POPIA requirements. Although patient identifiers are captured in the system, data for

purposes of this research project will be extracted, delinked from any patient identifiers and will rely on the ICS system-generated patient number.

Data for monitoring and evaluation of the intervention will be de-identified at the point of data collection.

- The investigator team providing support for monitoring and evaluation of the demand creation strategies will not have access to any information that will identify the individual patients. Further, the investigators will not have access to any patient files. The ICS-generated patient number will serve as the unique study number in the data set.
- GP practices will be assigned a study practice number.
- Field workers and the GPs will collect data that will be used for monitoring and evaluation. This data will be documented on routine systems and study-specific forms that will not contain any information that can identify individual patients.
- After the study consent form is signed, individuals that attend the GP practice for HTS will be given a unique study identifier through ICS. Data collected for this study will be de-identified and cannot be fully anonymised because GPs need to verify whether individuals tested for HIV and were linked to care if tested HIV positive. The link between patient IDs and unique study numbers will be kept by the GP in a locked cabinet at the selected GP practices. In addition, the links will be kept separate from other study documents to reduce the chances of linking the unique study number to the patient's file number.
- In addition to the data being de-identified, we will also aggregate findings when reporting (i.e., no individual cases will be reported on).

The study databases resulting from data collection will not contain identifying information and will be organized by assigned study ID numbers. Databases will be stored on password- protected computers. All database files will also be password-protected, and only study staff will have access to the files. Hard copy documentation will be stored in locked cabinets (at the GP practice overseen by the implementing partner). On completion of the final dataset(s), hard copies of

study documentation will be scanned. The hard copies will then be destroyed, and the electronic copies of the originals will be kept for six years after study completion before destroying them.

3.8 Study monitoring

The study sponsor, University of Pennsylvania (UPenn) may conduct monitoring or auditing of study activities to ensure the scientific integrity of the study and to ensure the rights and protection of study participants. Monitoring and auditing activities may be conducted by:

- Staff ("internal")
- Authorized representatives of UPenn

Monitoring or auditing may be performed by means of on-site visits or through other communications such as telephone calls or written correspondence. The visits will be scheduled at mutually agreeable times. During the visit, any study-related materials may be reviewed and the Investigator along with study staff should be available for discussion of findings. The study may also be subject to inspection by regulatory authorities (national or foreign) as well as the IECs/IRBs to review compliance and regulatory requirements.

4. STUDY TIMELINE

The study duration will be approximately 10 months. Table 2 below provides approximate time estimates allocated to each aspect of this research study. The final timeline will vary based on factors outside investigator control (i.e. ethics; other approvals).

Table 2: Study timeline

	2021			2022		
	Q2	Q3	Q4	Q1	Q2	Q3
Finalisation of protocol	X					
Sub-award process	X	X				
Ethics (Wits HREC, UPenn & BU)	X	X	X			
District Approval/Letter of support		X	X			
Randomisation sequence	X	X				
Protocol training		X				
Prototyping of brochures			X			
Data collection				X	X	
Data analysis					X	X
Report writing & dissemination					X	X

5. FUNDING SOURCES

5.1 Funding source

The GPCC is co-funded by the Gauteng Department of Health (funding for HIV test kits, HIV-related medicines and HIV-related laboratory services). Implementation of the GPCC (network management services, supply chain management and distribution services, remuneration of the contracted service providers for services rendered (HIV testing services, HIV management, dispensing)) are funded by a sub-award under Anova Health Institute under their USAID/PEPFAR APACE grant (Agreement Number 72067418CA00023). The proposed research grant will overlay the existing GPCC investment with support from the UPenn through Indlela (INV008318) to conduct the proposed study.

5.2 Conflict of interest

There are no known conflicts of interest.

6. DISSEMINATION PLAN

Presentation and publication of the results of this study will be governed by the Health Economics and Epidemiology Research Office (HE^{2}RO), Foundation for Professional Development, University of Pennsylvania and Boston University. For publication of the main findings of this study, the investigators will be considered as authors. Other key contributors to the research will be acknowledged or included depending on substantive contribution.

Subsequent secondary analyses and manuscripts will be agreed upon by the study investigators and in accordance with HE^{2}RO's publication policies. All scientific products such as abstracts, posters, presentations and manuscripts will undergo the HE^{2}RO's scientific clearance process. We will provide feedback to the participating GPs through the routine clinical review meetings (monthly virtual meetings with all GPs contracted onto the GPCC) and at district management and partner meetings. The GPCC team will share the study findings with practice staff at all participating GPs.

7. REFERENCES

1. UNAIDS. Data 2020. Program HIV/AIDS [Internet]. 2020;1–248. Available from: https://www.unaids.org/en/resources/documents/2020/unaids-data%0Ahttp://www.unaids.org/sites/default/files/media_asset/20170720_Data_book_2017_en.pdf
2. Lindenberg S, Papies EK. Two kinds of nudging and the power of cues: Shifting salience of alternatives and shifting salience of goals. Int Rev Environ Resour Econ. 2019;13(3–4):229–63.

8. APPENDICES

Appendix 1: Healthy lifestyle screen brochure
Appendix 2: Recipient of care voucher
Appendix 3: YCN standard of care brochure
Appendix 4: YCN field worker script for recruitment
Appendix 5: Distribution log
Appendix 6: Screening and enrolment log
Appendix 7: GPCC HTS consent form
Appendix 8: Participant information sheet
Appendix 9: Participant written informed consent form
