# Supplementary material for: Applying behavioural economics principles to increase demand for free HIV testing services at private doctor-led clinics in Johannesburg, South Africa: A randomised controlled trial
Source: PLOS Glob Public Health. 2024 Aug 6;4(8):e0003465. doi: 10.1371/journal.pgph.0003465 (PMC11302913; doi:10.1371/journal.pgph.0003465)
Supplement: Attachment — Submitted filename: PGPH-D-23-01678 Responses to reviewers.docx.pdf [file pgph.0003465.s007.pdf]

Indlela@heroza.org
Linkedin

INDLELA BEHAVIOURAL INSIGHTS FOR BETTER HEALTH @ Health Economics and Epidemiology Research Office HE²RO Wits Health Consortium University of the Witwatersrand

PLOS Global Public Health
United Kingdom

PLOS Global Health Reference Number: PGPH-D-23-01678
Re: Response to Reviewers

Dear Dr. Catherine Kyobutungi and Prof. Madhukar Pai

We appreciate the feedback received from you and the reviewers on our manuscript entitled "*Applying behavioural economics principles to increase demand for free HIV testing services at private doctor-led clinics in Johannesburg, South Africa: A randomised controlled trial*". Below is a point by point response to the Reviewer comments:

Reviewer 1

1. P4L57-60: You will need to explain to the reader what the 95-95-95 target is (including a citation), as anybody not familiar with the field will struggle to understand.

   Thank you for this reminder. We provided a brief description of the UNAIDS 95-95-95 targets and this reads as follows: "*These goals are aimed at ending the AIDS pandemic by ensuring that 95% of all people living with HIV know their HIV status, 95% of all people diagnosed with HIV infection receive antiretroviral therapy (ART), and 95% of all people receiving ART are virally suppressed (UNAIDS, 2024)*".

2. P4L62-65: What is considered to be low? Also, are these groups specifically vulnerable?

   We added text in the manuscript to clarify that high HIV testing coverage in South Africa hides large disparities in coverage for certain populations including gay men and other men who have sex with men, female sex workers and people who inject drugs. The revised text reads as follows: "*However, the overall success of the HIV testing programs hides disparities in testing coverage; rates remain lower in certain populations at increased vulnerability to risk of HIV infection including gay men and other men who have sex with men, female sex workers and people who inject drugs*[32]"

3. P4L77-79: Given that this is for an international audience, you will have to provide more elaboration here [on the NHI].

Thank you to the reviewer for highlighting that more context is needed for those who are not familiar with South Africa and their policies. The NHI Bill was recently signed into law on 15 May 2024. We revised the language to refer to the NHI Bill as a law instead of a proposal. The following information was included: "*The NHI Bill was signed into law on 15 May 2024 and aims to provide universal health coverage to all South Africans through a single pool of funding for private and public healthcare providers*[10]."

4. P4L78-80: However, there are still limited studies that evaluate this type of model in low- and middle-income countries (LMICs) and even fewer studies evaluating demand creation for these services." Do you have a reference for this?

   There are few studies that evaluate a model similar to the NHI in resource constrained countries. We added some references from studies conducted in low-and middle income countries.

5. P5L85-94: Elaborate on "present bias" for the reader. Also, perhaps elaborate on the studies you cite here. What do they say about present bias being a barrier to testing? Overall comment of introduction/background: This is a behavioural study, but there is very little discussion of the behavioural principles that you are studying and the evidence on these principles or how they tend to affect health outcomes (i.e. framing effects, present bias). Please build on this.

   Thank you for this comment and the opportunity to rectify this oversight. We included the following detail on P5L86-99 that reads as follows: "*In healthy individuals, the benefits of engaging in HIV prevention and treatment are not immediate and individuals tend to postpone accessing these services. In a study conducted in Uganda with PLHIV on ART, 36% were classified as present-biased as they preferred an immediate hypothetical reward over a large reward that would be available a year later*[17]. *In the same study, the present-biased participants were also less likely to adhere to ART. In addition to present bias, the framing of behavioural messages or interventions determines the acceptability and uptake of the intervention. Another study in rural Uganda that assessed the effectiveness of incentive strategies to promote HIV testing among men used various behaviourally informed framing styles such as loss framing in which individuals were offered a reward with the condition that they perform the desired behaviour*[18]. *Similar studies have been conducted in Uganda, Tanzania and other countries using behaviourally informed*

interventions such as small incentives and planning prompts to improve HIV testing and have shown the potential of low-cost interventions to increase the uptake of HIV testing services[19-22]".

6. P6L125-129: Please elaborate on the overall and health sector context of Johannesburg for international readers.

   We added the following sentence to briefly elaborate on the health sector context of Johannesburg: "*Johannesburg is South Africa's most populous city, with 5.5 million residents, most (84%) of which are uninsured for healthcare services and accessing public health facilities*[24]. In 2020, the HIV prevalence was 13% in Johannesburg with the highest incidence recorded among females compared to males of all age groups[25]."

7. P7L156-161: How are these [loss aversion and endowment effect] linked to the pamphlet (more detail is necessary), and how will they overcome the barriers.

   We added some text to clarify how the BE principles are linked to the brochure and how they address barriers to HIV testing. The added text reads as follows: "*Our hypothesis was that individuals who receive the voucher brochure will try to avoid losing its value by redeeming it for an HIV test before it expires. HIV testing is typically provided at no cost at public health clinics in South Africa but is typically not free in the private sector. Attaching a monetary value to the HIV test increases the perceived value of a service that many South Africans may consider low value because it is offered free of charge.*"

8. P8L169-172: small block sizes (15) to ensure uniform distribution of the 3 brochures in each of 3 languages- Sorry, it's unclear what this means. Was randomization done at an individual level?

   Thank you for highlighting that this description was not clear. The brochures were individually randomised. Participants had the option to get a brochure in any of the three study languages including English, IsiZulu and SeSotho. To manage brochure distribution across the three languages we used small block sizes (15) to ensure that there was equal assignment across the study arms (brochures) irrespective of which language was chosen. We revised L177-180 to capture this clarification, the sentence reads as follows: "*In order to ensure that the randomisation was balanced across the different language options, we used small block sizes (15) to ensure that there was equal assignment across the study*

arms (brochures) and uniform distribution of the 3 brochures in each of 3 languages." L182-185 further explains the distribution of the brochures across the 3 languages: "*During distribution, the individually randomised brochures were ordered into three language packs and fieldworkers distributed the brochures to participants in the order of the language packs depending on the participants' language preference.*"

9. P8L187-188: It's not exactly clear to me why recipients of the pamphlets would need to bring the pamphlet with them to their clinic visits, other than with the "voucher model". How do you know that people weren't incentivised to go to the clinic and did not bring their pamphlet along? I'm sure this was controlled for, but it's not coming out clearly in the write-up.

   Thank you for this comment. The main reason for asking participants to bring the brochures (any form) was for proper allocation to the study arms. However, even those who did not bring the brochures to the clinic but were able to describe details of the specific brochure that they received were allocated to the specific arm and enrolled in the study. If participants did not have a brochure and were not able to describe any of the study brochures, they received standard routine services at the clinic and were not enrolled in the study. At the point of brochure distribution, participants were allowed to take more than one brochure and share these with individuals in their circles. All participants visiting the private-led clinics during the study period had access to an HIV test, whether as part of the study or through routine clinic services and screening before enrolment ensured that participants were correctly assigned to the study or routine clinic services.

10. Methodology overall: Did you do any piloting work that showed that present bias was a problem in the context of your clinics? Or what the main barriers to access in this catchment area are?

    During preparation for this project, we conducted an extensive literature search on HIV care in South Africa, demand creation for HIV testing services and the use of behavioural science in HIV testing programmes. The main barriers to accessing HIV services among others included people focussing on present benefits than the future when making health decisions, and some overly confident on their ability to use one form of HIV prevention thereby seeing less value in HIV testing as bargaining future benefits with present[17,18]. From consultations with the implementing partner that was providing HIV services in the

catchment area, barriers such as lack of knowledge about the HIV testing services provided at the private doctor-led clinics, lack of interest in HIV testing owing to perceptions of minimal risk to infection, inability to foresee the benefits of HIV testing were noted. This contextually relevant information supported what we found in the literature.

Although we did not conduct a contextual inquiry pilot to identify the behavioural barriers and biases, we participated in a number of meetings with the programme implementers that were conducted during the early stages of co-designing the intervention. From these meetings, we gathered from discussion with programme staff that present bias was likely among the reasons impeding access to HIV services.

11. P12L263-266: Among participants receiving brochures, 448 (3.9%) individuals presented at a private doctor-led clinic for services within 4 months and 50.7% of those presenting were males (see supplementary table 1). Is this your final sample size? Is this enough to have power? Also, what are the demographic differences between the group that used the pamphlets, and the ones that did not (e.g. the whole 11000 sample)?

    The sample size was calculated based on the number of brochures to be distributed as indicated in the last paragraph of the "randomisation and data collection" section. The total sample size was 12, 000 and this would ensure that there is 80% power to detect a difference of at least 2 percentage points in attendance at private doctor-led clinics. The number presented in the paragraph mentioned here, is just the number of people presenting at the clinic following exposure to any of the study brochures. The demographic differences between the group that presented at the clinic and those that did not are presented in the supplementary material (Supplementary Table 1: Proportion of individuals presenting at the GP practice over the total number of brochures distributed by study arm). About 2% of all males (6774) and females (4601) who received a brochure presented at the private doctor-led clinic for HTS.

12. P14L292-296: Establishing the effectiveness of the specific behavioural insights used to inform the intervention brochures is important in understanding the overall potential of behavioural economics in improving HIV outcomes. This point does not flow from the previous point on the visibility of clinics, and it's a bit out of place and unsubstantiated as it stands here.

Thank you for this point. We added a sentence to connect the last sentence. The added sentence reads as follows: "*These findings highlight the various factors influencing the uptake of HIV services at private doctor-led clinics.*"

13. P15L300-301: This should be in the results section, not the discussion section. The rest of the paragraph is very interesting and should stay in the discussion section and refer to this finding in the results. I wonder if "interestingly" is an appropriate word to use in a journal context.

    Thank you for the comment. These results are already included in the results section. We have removed this sentence from the discussion section.

Reviewer 2

14. P6L134-137: There is opportunity to provide further information on site characteristics because they emerge as a key component of the results. For example, high visibility, and central location only appear in the results section, with little background into how these are defined.

    Thank you for highlighting this oversight. We included the following sentence to the site characteristics: "*Two of the clinics had branded gazebos erected in the clinic for the purposes of HIV testing, whereas others only offered clinic-based testing (in which testing was done inside the clinic building). Branded gazebos increased the clinic's visibility.*"

15. P9L195-200: Please clarify how study staff knew the arm to which participants were assigned if they showed up without the brochure or envelope.

    We reference L195-197 and have added text in L198-200 to clarify this information: *"Individuals demonstrating exposure (presenting a study brochure) or self-reporting exposure (reporting having received a brochure from a fieldworker or other contact but not having the brochure present) were invited to participate in the study. Individuals self reporting exposure were asked to describe the brochure they were exposed to and shown the described brochure to confirm exposure."*

16. P10L229-233: Please clarify if this means that everyone had at least 4 months to make the clinic visit. If the "follow-up time" depended on when one received the brochure relative to the end date of data collection, please describe when the distribution of brochures stopped for each of the clinics. It is helpful to know this for putting the results in context (especially

the secondary analysis where comparisons are made at the clinic level): if the sample size for Clinic X was reached earlier than Clinic Y, could that have given participants from Clinic X more opportunity to make the clinic visit?

Thank you for giving us the opportunity to clarify this information. The duration to present at the clinic depended on when the participant received the brochure. Data collection took place over 4 months across all clinics. We added the following sentence to further clarify this point: "*The distribution of brochures at all clinics ended at least 2 weeks before the end date of the data collection period at the private doctor-led clinics ended. This method allowed time for participants who had received brochures to present at the clinic. The period for presenting at the clinic for HTS was contingent upon the receipt of the brochure and ranged from a minimum of 2 weeks to a maximum of 4 months.*"

17. P15L318-323: However, we found that distributing brochures in these locations outside of traditional healthcare facilities reached more men than women across all clinic locations. It is not entirely clear how this finding is to be interpreted. Could this be an artefact of how study staff distributed the brochures since they had some control over who they approached to give the brochure?

    Thank you for this interesting question. The results cannot be attributed to a single factor. We added text on the discussion section to clarify this information: "*Several factors could have influenced the higher uptake of HTS among males. The novel approach to promoting HIV testing in private, doctor-led clinics may have been particularly appealing to men. The distribution sites were also areas where men were more likely to pass through as they go or return from work. However, we also acknowledge that the results may be influenced by the manner in which the brochures were distributed and who was more willing to accept the brochures.*"

18. Brochures: The brochures suggest that participants had to send a WhatsApp message to book an appointment. If so, were there any considerations for people without cell phones?

    Thank you for raising this point about phone ownership. WhatsApp booking was mainly for those who wished to make an appointment or find other clinics they could go to, however, all brochures included the text "Walk-ins welcome" to accommodate participants who might not have had access to WhatsApp or a phone. This text was added to P7L161-164.

Indlela@heroza.org
Linkedin

INDLELA BEHAVIOURAL INSIGHTS FOR BETTER HEALTH @ Health Economics and Epidemiology Research Office HE²RO Wits Health Consortium University of the Witwatersrand

Journal requirements

1. Please review your reference list to ensure that it is complete and correct. If you have cited papers that have been retracted, please include the rationale for doing so in the manuscript text, or remove these references and replace them with relevant current references.

   We have addressed the reviewers comments and added more references which are also included in the reference list. We also reviewed the reference list for completeness and accuracy, no references were removed from the list.

2. Please ensure you have included the registration number for the clinical trial referenced in the manuscript.

   The trial registration number is included in P3L51 of the manuscript: "*SANCTR (South African Clinical Trials Register) registration number: DOH-27-032022-9053).*"

3. Please provide separate figure files in .tif or .eps format only and remove any figures embedded in your manuscript file. Please also ensure all files are under our size limit of 10MB.

   The following figures that were embedded in the manuscript were removed and submitted as a separate file named "Figures and tables: manuscript" and include the following:

   - **Figure 1:** CONSORT diagram describing the total number of brochures received by participants across the three study arms (n=11,471)
   - **Table 1:** Characteristics of participants who received a brochure to attend HTS at a private clinic between 24 January 2022 to 15 April 2022
   - **Figure 2:** Proportion of individuals presenting at the private doctor-led clinic by study arm
   - **Table 2:** Effect of intervention on odds of presenting at the GP practice with study arm

4. We have noticed that you have uploaded Supporting Information files, but you have not included a list of legends. Please add a full list of legends for your Supporting Information files after the references list.

   We included a list of all the supporting documents submitted and these are included below:

   - *Supplementary material*

- *Completed CONSORT checklist*
- *Study protocol version 2*
- *University of the Witwatersrand ethics approval letter*
- *University of the Witwatersrand protocol amendment ethics approval letter*
- *University of Pennsylvania ethics approval letter*
- *Boston University IRB reliance outcomes agreement letter*
- *Data underlying the findings*

5. In the online submission form, you indicated that "Data is available upon reasonable request". All PLOS journals now require all data underlying the findings described in their manuscript to be freely available to other researchers, either 1. In a public repository, 2. Within the manuscript itself, or 3. Uploaded as supplementary information.

   All data underlying the findings is available on the OSF data repository and the section on data availability has been updated to read as follows: "*Data underlying the findings is available on the Open Science Framework data repository on https://osf.io/uqn9c/?view_only=cd18ae5654054b309a117d63be4fc652.*"

We made grammatical and text edits throughout the document.

Thank you for considering our work.

Yours Sincerely,

Simamkele Bokolo

Position: Researcher

Email: sbokolo@heroza.org

Cellphone: +27 (0) 73 829 7261
